# Adjunctive bright light therapy for treating bipolar depression: A systematic review and meta‐analysis of randomized controlled trials

**DOI:** 10.1002/brb3.1876

**Published:** 2020-10-09

**Authors:** Hirofumi Hirakawa, Takeshi Terao, Masaaki Muronaga, Nobuyoshi Ishii

**Affiliations:** ^1^ Department of Neuropsychiatry Faculty of Medicine Oita University Oita Japan

**Keywords:** bipolar disorder, bright light therapy, meta‐analysis, randomized control trials

## Abstract

**Objectives:**

Bright light therapy (BLT) was reported as an effective adjunctive treatment option for bipolar disorder. Previous meta‐analytic study showed that augmentation treatment with light therapy significantly decreased the severity of bipolar depression. However, most of included studies were case–control studies and several of them focused on BLT that was provided in combination with sleep deprivation therapy.

**Methods:**

In this meta‐analysis, we used several electronic databases to search the studies and included only randomized controlled trial (RCT) studies to compare BLT with control experimental groups for treating bipolar depression with pharmacological treatment to clarify the adjunctive efficacy of BLT. We searched the databases of EMBASE, MEDLINE, Scopus, The Cochrane Central Register of Controlled Trials, the Cumulative Index to Nursing and Allied Health Literature, and Clinicaltrials.gov for studies published in English until September 19, 2019. Two researchers conducted the literature screening, data extraction, and methodological quality assessment independently. The main outcome was the response rate and remission rate. We used the Review Manager 5.3 Software for the meta‐analysis.

**Results:**

Four trials with a total of 190 participants (intervention: 94, control: 96) with bipolar depression were evaluated to gauge the effects of light therapy. The meta‐analysis showed risk ratios of 1.78 (95% CI 1.24–2.56, *p* = .002; *I* ^2^ = 17%) demonstrating a significant effect of light therapy in the response rate of bipolar disorder. The meta‐analysis shows risk ratios of 2.03 (95% CI 0.48–8.59, *p* = .34; *I* ^2^ = 67%) demonstrating no significant effect of light therapy in the remission rate of patients with bipolar disorder. None of the articles reported any serious adverse effects. Manic switch rate was 1.1% in the light therapy group and 1.2% in the control group.

**Conclusions:**

Bright light therapy is an effective treatment for reducing depression symptoms among patients with bipolar depression.

## Graphical abstract

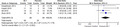

## 1 INTRODUCTION

Bipolar disorder is a chronic disorder characterized by fluctuations in mood state and energy, with the patients experiencing recurrent episodes of elevated mood and depression (Grande et al., 2016). Bipolar disorder, mainly diagnosed in young adulthood, leads to cognitive and functional impairment of daily life (Grande et al., 2016). It is a disabling illness due to its early onset and usually requires long‐term treatment. Mood stabilizers and atypical antipsychotics are the main pharmacological treatments of bipolar disorder. Specifically, lithium, one of the mood stabilizers, has been increasingly found to be effective in treating acute manic episodes, preventing relapses, and treating bipolar depression (Geddes & Miklowitz, 2013). Antidepressants are not recommended as monotherapy, and they result in a 15%–40% rate of manic switches during antidepressant drug treatment (Benedetti, 2018). In long‐term management, alternative nonpharmacological treatment approaches are required to stabilize patients’ moods.

Bright light therapy (BLT) as a treatment option was first suggested in association with the seasonal affective disorder (SAD; Rosenthal et al., 1984). Now, BLT is well‐known and has been used for treating not only seasonal affective disorder but also bipolar depression (Pail et al., 2011; Tseng et al., 2016). BLT was reported as an effective adjunctive treatment option for bipolar disorder (Hirakawa et al., 2019). Various mechanisms for the action of BLT have been proposed, including the modulation of circadian rhythms by regulating the suprachiasmatic nucleus, extension of the photoperiod, regulation of melatonin secretion, advancement of circadian rhythms, and interactions with serotonin (Murray et al., 2005; Pail et al., 2011). BLT is an effective, accepted, safe, nonpharmacological, low‐cost treatment and has a very favorable risk‐to‐benefit ratio for depressive disorders (Terman & Terman, 2005). BLT showed a lower risk of manic switches (2.3%) than antidepressants (15%–40%; Benedetti, 2018).

Previous meta‐analytic study showed that augmentation treatment with light therapy significantly decreased disease severity of bipolar depression (Tseng et al., 2016). However, this study had some limitations. First, the studies used were not randomized controlled trial (RCT), and most of the studies were case–control studies. Second, several studies focused on BLT administered in combination to sleep deprivation therapy. Third, articles were searched through only one electronic database (PubMed). In this meta‐analysis, we used several electronic databases to search the studies, and we included only RCT studies that compared BLT with control experimental groups to clarify the adjunctive efficacy of BLT in treating bipolar depression.

## 2 METHODS

### 2.1 Data sources

A Systematic Review was performed according to Preferred Reporting Items for Systematic Reviews and Meta‐Analyses (PRISMA) reporting guidelines. Research of current literature in English until September 19, 2019 was carried out in five electronic databases: EMBASE, MEDLINE, Scopus, The Cochrane Central Register of Controlled Trials (CENTRAL), and the Cumulative Index to Nursing and Allied Health Literature (CINAHL). We also manually searched the references used by the identified papers; additionally, unpublished and ongoing trials were searched on Clinical Trials (http://clinicaltrials.gov). The search was conducted by two independent authors (HH and MM), and was performed using the keywords “light therapy,” “phototherapy” and “bipolar disorder.” The titles and abstracts in connection with these articles were screened to determine whether they were potentially eligible for inclusion in this study. All reports that were not related to the application of light therapy in bipolar disorder were excluded. TT performed checks to ensure quality and consistency of the assessment and made the final judgment and decision. In cases where there were unavailable or unmentioned data for a published article, HH contacted the authors to acquire the original data.

### 2.2 Inclusion and exclusion criteria

Studies’ inclusion criteria were as follows: (a) RCT that compared BLT with control experimental groups (dim light or negative ion generators) as an adjunctive treatment for the acute‐phase treatment of adults (aged 18 years or older) of both sexes, with a primary diagnosis of bipolar disorder according to standard operationalized diagnostic criteria, (b) Evaluated by standardized scales for assessing depression, such as the Hamilton Depression Rating Scale (HAM‐D), the Hamilton Depression Rating Scale with Atypical Depression Supplement (SIGH‐ADS), or the Montgomery‐Åsberg Depression Rating Scale (MADRS), (c) Light therapy needed to be identified as the experimental group intervention. We did not restrict the different conditions, such as illumination and exposure times of the light therapy.

Also, studies’ exclusion criteria were as follows: (a) Studies of individuals diagnosed with depression or seasonal affective disorder. (b) Studies which included other treatment options such as those in which light therapy was provided in combination with sleep deprivation therapy to evaluate the effect of adjunctive light therapy itself.

### 2.3 Outcome measures

The main outcome was the response rate and remission rate (response defined as 50% or greater reduction in depression severity on the HAM‐D or SIGH‐ADS or MADRS and remission defined as a SIGH‐ADS score less than 8 or reduction to 7 points in HAM‐D and 9 points in MADRS). Secondary outcomes were occurrence of adverse events such as manic switches and acceptability (rate of dropouts for any reasons). The data were entered into the Cochrane Collaboration's Review Manager Analysis Version 5.3 statistical software for meta‐analysis and preparation of graphical figures.

### 2.4 Assessment of risk of bias

We used Cochrane collaboration's risk of bias tool to evaluate the potential risk of bias for each included study (Higgins et al., 2011). The tool included the following seven factors: random sequence generation, allocation concealment, blinding of participants and personnel, blinding of outcome assessment, incomplete outcome data, selective outcome reporting, and other possible sources of biases. For each domain, we made a judgment with “yes,” “no,” or “unclear,” and used the Review Manager 5.3 to analyze and display the results, which were interpreted in terms of the findings regarding the risk of bias. A funnel plot was generated to visually inspect publication bias.

### 2.5 Statistical analyses

To evaluate the response rate and remission rate for the light group in comparison to the control groups, we analyzed dichotomous outcomes using risk ratios and 95% confidence intervals (CIs). A significance level of 5% was used for all statistical tests. For the analysis, we used the Mantel–Haenszel method to estimate the significance level. All meta‐analyses were carried out using a random‐effects model. The *I* ^2^ test statistic was used to determine the extent of variation between sample estimates with values ranging from 0% to 100% to assess heterogeneity.

### 2.6 Ethical approval

Ethical approval was waived because this study did not involve any human participants or animals.

## 3 RESULTS

### 3.1 Study identification and selection

**FIGURE 1:**
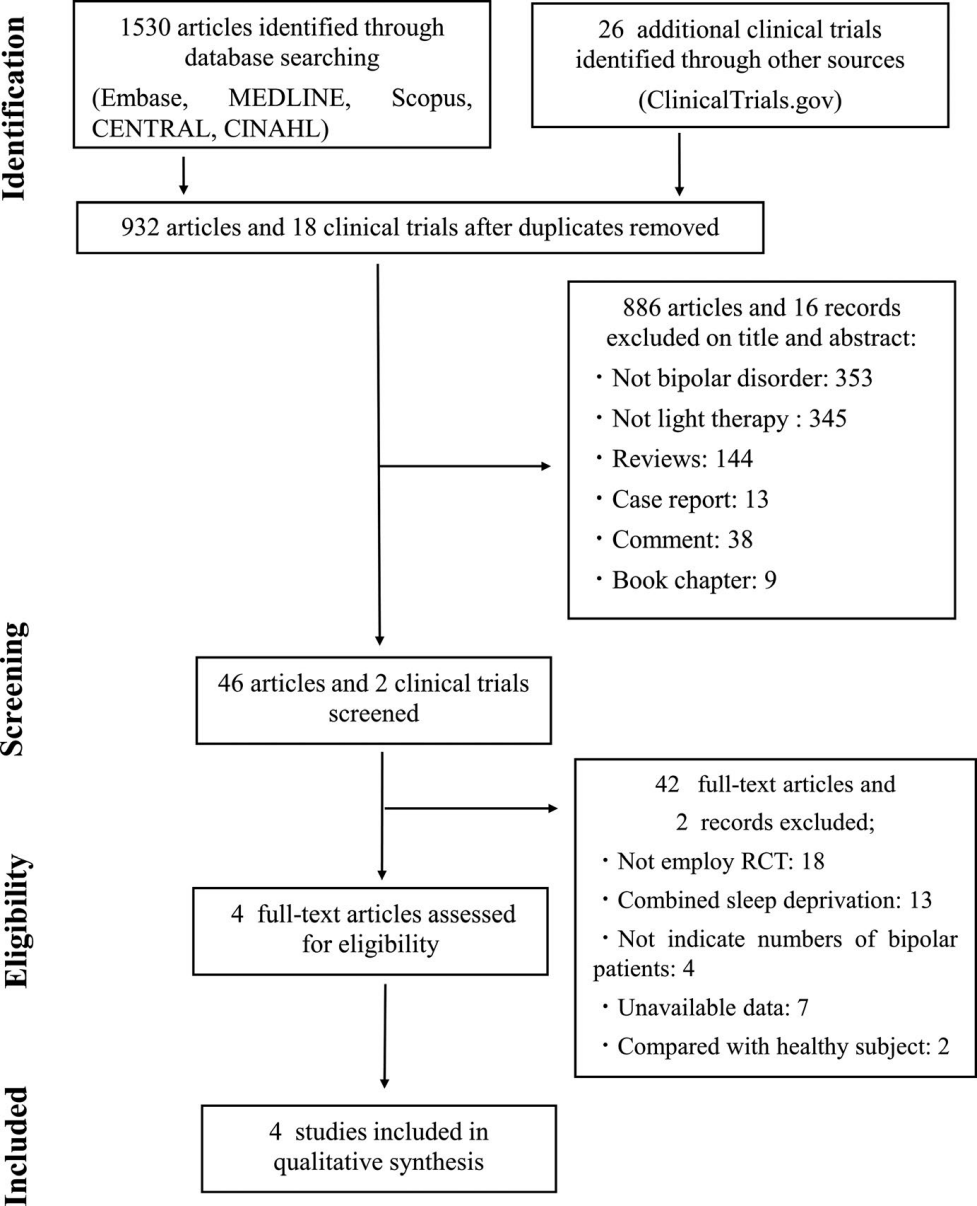
PRISMA flow chart of the study. Initially, 1,530 studies were found using the search terms from five databases and 26 clinical trials identified on the ClinicalTrials.gov website. Finally, we included 4 studies for final analyses

Initially, 1,530 studies and 26 clinical trials were identified through the usage of the search terms in the five databases and the ClinicalTrials.gov website, respectively. After screening the titles and abstracts and removing irrelevant articles and duplicates, 46 articles and two clinical trials were included for screening using the full‐text. A total of 42 studies were excluded because they: were not RCT in design (*n* = 18; Bauer et al., 1994; Benedetti, et al., 2014, 2009, 2014, 2009; Bria et al., 2012; Dallaspezia et al., 2018; Deltito et al., 1991; Franchini et al., 2009; Geoffroy et al., 2018; Krauss et al., 1992; Kripke et al., 1983; Kusumi et al., 1995; Leibenluft et al., 1995; Mazaheri Nazari Far et al., 2011; Mazza et al., 2016; Melloni et al., 2016; Papatheodorou & Kutcher, 1995; Sit et al., 2007; Wehr et al., 1985), combined sleep deprivation with light therapy (*n* = 13; Benedetti et al., 2003, 2005, 2007, 2009, 2010, 2014; Chojnacka, et al., 2016; Colombo et al., 2000; Sikkens et al., 2019; Suzuki et al., 2016, 2018; Vai et al., 2015; Wu et al., 2009), included patients with unipolar depression or depression and did not indicate the number of patients with bipolar disorder (*n* = 4; Beauchemin & Hays, 1997; Benedetti, et al., 2003; Chojnacka, et al., 2016; Roecklein et al., 2012), and compared with healthy subjects (*n* = 2; Ritter et al., 2019; Whalley et al., 1991), had unavailable data (NCT01431573, NCT02088580, NCT03396744, NCT02176824, NCT00590265, NCT03679962, ChiCTR‐INR‐17013250; *n* = 7). Finally, we included 4 studies (Dauphinais et al., 2012; Sit et al., 2018; Yorguner Kupeli et al., 2018; Zhou et al., 2018) for final analyses (see Figure 1 for the literature screening flow chart).

### 3.2 Characteristics of included studies

The detailed characteristics of the included studies are shown in Table 1. The four included studies contained a total of 190 patients (Dauphinais et al., 2012; Sit et al., 2018; Yorguner Kupeli et al., 2018; Zhou et al., 2018). All studies included a mix of bipolar I and bipolar II patients, two studies mentioned about the numbers of type of bipolar (Sit et al., 2018; Yorguner Kupeli et al., 2018), the others were not (Dauphinais et al., 2012; Zhou et al., 2018). The numbers of participants of intervention group were 94 (male: 37, female: 57) and of control group were 119 (male: 57, female: 62). Mean age was 40.6 years of all participants, 40.3 years in the light group and 40.9 years in the control group). The conditions of the intervention group varied, such as light intensity (varying range from 5,000 to 10,000 lux), the color temperature of light (4,000 to 10,000 Kelvin), and exposure time (varying range from 15 to 60 min). One study did not mention anything about the color temperature of light (Yorguner Kupeli et al., 2018). The duration of intervention time also varied from 2 to 8 weeks. As a control experimental group, three studies used dim red light (Sit et al., 2018; Yorguner Kupeli et al., 2018; Zhou et al., 2018) and one study used negative ion generators (Dauphinais et al., 2012). The outcome measures were different. Three studies used the HAM‐D (Sit et al., 2018; Yorguner Kupeli et al., 2018; Zhou et al., 2018), two studies used SIGH‐ADS (Dauphinais et al., 2012; Sit et al., 2018), and two other studies used MADRS (Dauphinais et al., 2012; Yorguner Kupeli et al., 2018). All studies were designed as adjunctive therapy of BLT and most of the studies included patients treated with mood stabilizers or antidepressants. The details of study characteristic are described in Table 1.

**TABLE 1:** Study characteristics

Study	Study details													
	Diagnosis and criteria	Main outcome measures	Length of trial	Study design	Number of participants of intervention group and control group	Intervention details	Number of female	Mean age (*SD*)	Type of BP	Medication of MS, AD, AP	Response rate	Remission rate	Manic switch rate	Dropout rate
Sit et al. (2018)	BP I or BP II depression DSM‐IV	HAM‐D (21‐items), SIGH‐ADS	6 week	RCT (two‐arm) double‐blind (participants and assessor)	Intervention *n* = 23	7,000 lux 4,000 K White light 15–60 min Midday	14 (60.9%)	45.7 (14.3)	BP I:13 (56.5%), BP II :10 (43.5%)	MS < AC: 12 (52.2%), Li:6 (26.1%)> AD:17 (73.9%) AP: 14 (60.9%)	16/22 72.7%	15/22 68.2%	0%	1/23 4.34%
					Control *n* = 23	50 lux Red light 15–60 min Midday	17 (73.9%)	43.7 (15.0)	BP I:18 (78.3%), BP II :5 (21.7%)	MS < AC: 15 (65.2%), Li: 4 (23%)> AD:19 (82.6%) AP:17 (73.9%)	9/18 50.0%	4/18 22.2%	0%	5/23 21.7%
Zhou et al. (2018)	Bipolar disorder depression DSM‐IV	HAM‐D (17‐items) QIDS‐SR16	2 week	RCT (two‐arm) Single‐blind (participants)	Intervention *n* = 33	5,000 lux 10,000 K Bright light 60min Morning	20 (60.6%)	35.09 (14.19)	NA	MS:33 (100%)	26/33 78.8%	‐	0%	4/37 10.8%
					Control *n* = 30	Less than 100lux Dim red light Morning	14 (46.7%)	39.73 (13.53)	NA	MS:30 (100%)	13/30 43.3%	‐	0%	7/37 18.9%
Yorguner Kupeli et al. (2018)	BP I or BP II depression DSM‐IV	HAM‐D, MADRS	2 week	RCT (two‐arm) Single‐blind (participants)	Intervention *n* = 16	10,000 lux Bright light 30min Morning	10 (62.5%)	42.1 (9.1)	BP I:10 (62.5%), BP II :6 (37.5%)	MS < Li:9 (56.2%), LTG:2 (12.5%), VPA: 3 (18.8%)> AD: 6 (37.5%) AP: 6 (37.5%)	11/16 68.8%	7/16 43.8%	0%	0/16 0%
					Control *n* = 16	Less than 500lux Dim light Morning	16 (100%)	37.1 (8.2)	BP I:7 (43.8%), BP II :9 (56.2%)	MS < Li:8 (50%), LTG:5 (31.3%), VPA: 3, (18.8%), CBZ:2(12.5%)> AD: 6 (37.5%) AP: 9 (56.3%)	2/16 12.5%	1/16 6.25%	0%	0/16 0%
Dauphinais et al. (2012)	BP I or BP II depression DSM‐IV	SIGH‐ADS, MADRS	8 week	RCT (three‐arm) Single‐blind (assessor)	Intervention *n* = 18	7,000 lux 4,000 K Bright light 7.5– 15 min Morning	13 (72.2%)	42.4 (12.4)	NA	NA	7/18 38.9%	2/18 11.1%	1/18 (hypomania) 5.5%	8/18 44.4%
					Control *n* = 20	Negative ion air Morning	15 (75%)	43.1 (16.0)	NA	NA	5/20 25%	5/20 25%	1/20 (hypomania) 5%	9/20 45%

Abbreviations: AC, Anticonvulsants; AD, Antidepressants; AP, Antipsychotics; BP, Bipolar disorder; CBZ, Carbamazepine; DSM, Diagnostic and Statistical Manual of Mental Disorders; HAM‐D, Hamilton Depression Rating Scale; Li, Lithium; LTG, Lamotrigine; MADRS, Montgomery–Åsberg Depression Rating Scale; MS, Mood Stabilizer; NA, Not Available; QIDS‐SR16, The 16‐item Quick Inventory of Depressive Symptomatology Self‐report; RCT, Randomized controlled trial; SIGH‐ADS, Hamilton Depression Rating Scale with Atypical Depression Supplement; VPA, Valproate.

### 3.3 Risk of bias in included studies

**FIGURE 2:**
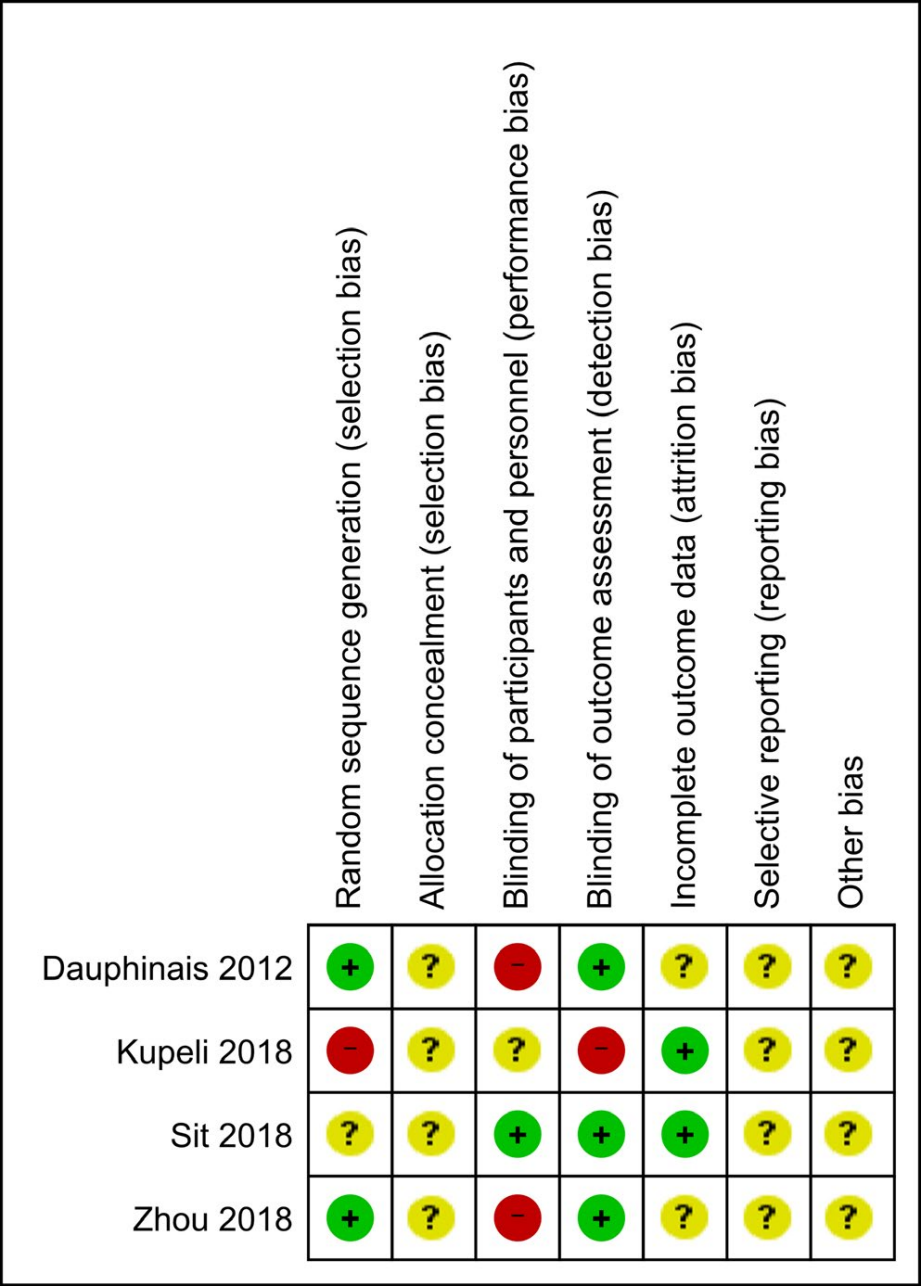
Quality assessments of included studies. The risk of bias assessments using the Cochrane Risk of Bias Tool was summarized

**FIGURE 3:**
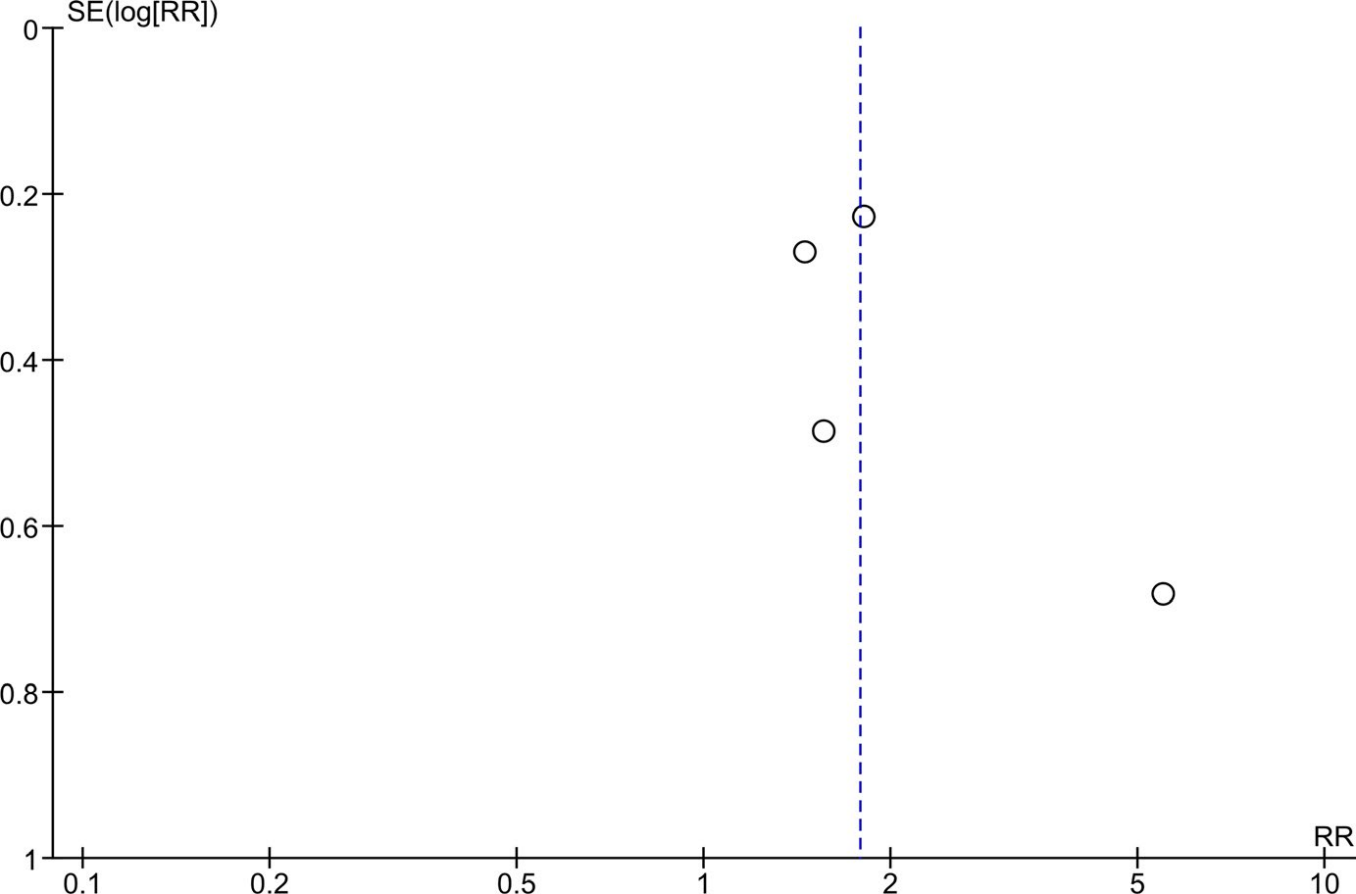
Funnel plot showing publication bias. A funnel plot was generated using the 4 studies included in the meta‐analysis. There appears to be asymmetry about the funnel, suggesting possibility of publication bias

The risk of bias assessments using the Cochrane Risk of Bias Tool is summarized in Figure 2. One study was judged to have a high risk of randomization methods (patients were randomized according to their admission order; Yorguner Kupeli et al., 2018). A funnel plot (Figure 3) was generated using the four studies included in the meta‐analysis. Our meta‐analysis included only four studies (white circles), and there appears to be asymmetry about the funnel, suggesting possibility of publication bias.

### 3.4 Findings of light therapy on bipolar depression

**FIGURE 4:**
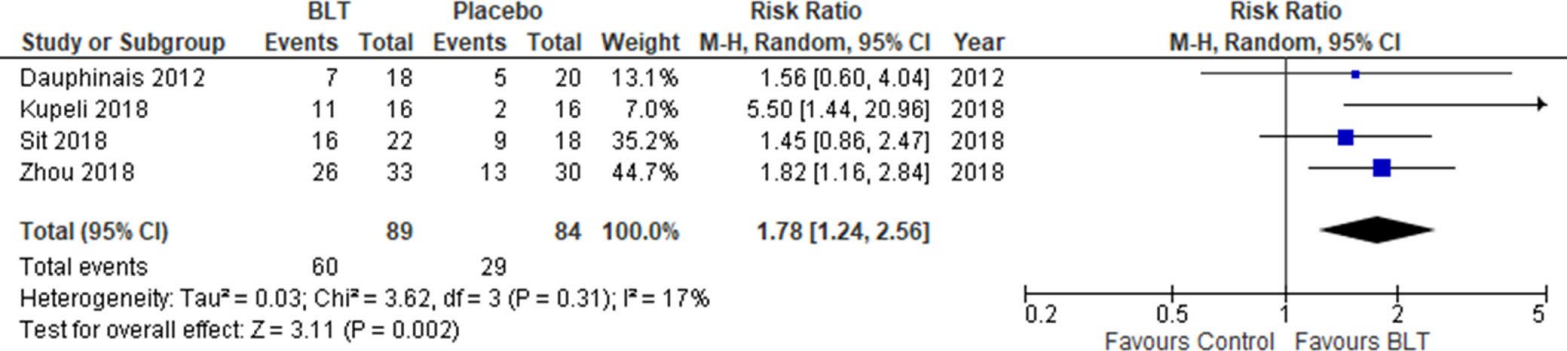
Effect of light therapy for bipolar depression (response rate). The meta‐analysis shows risk ratios of 1.78 (95% CI 1.24–2.56, *p* = .002; *I* ^2^ = 17%) demonstrating a significant effect of light therapy in the response rate of patients with bipolar disorder

**FIGURE 5:**
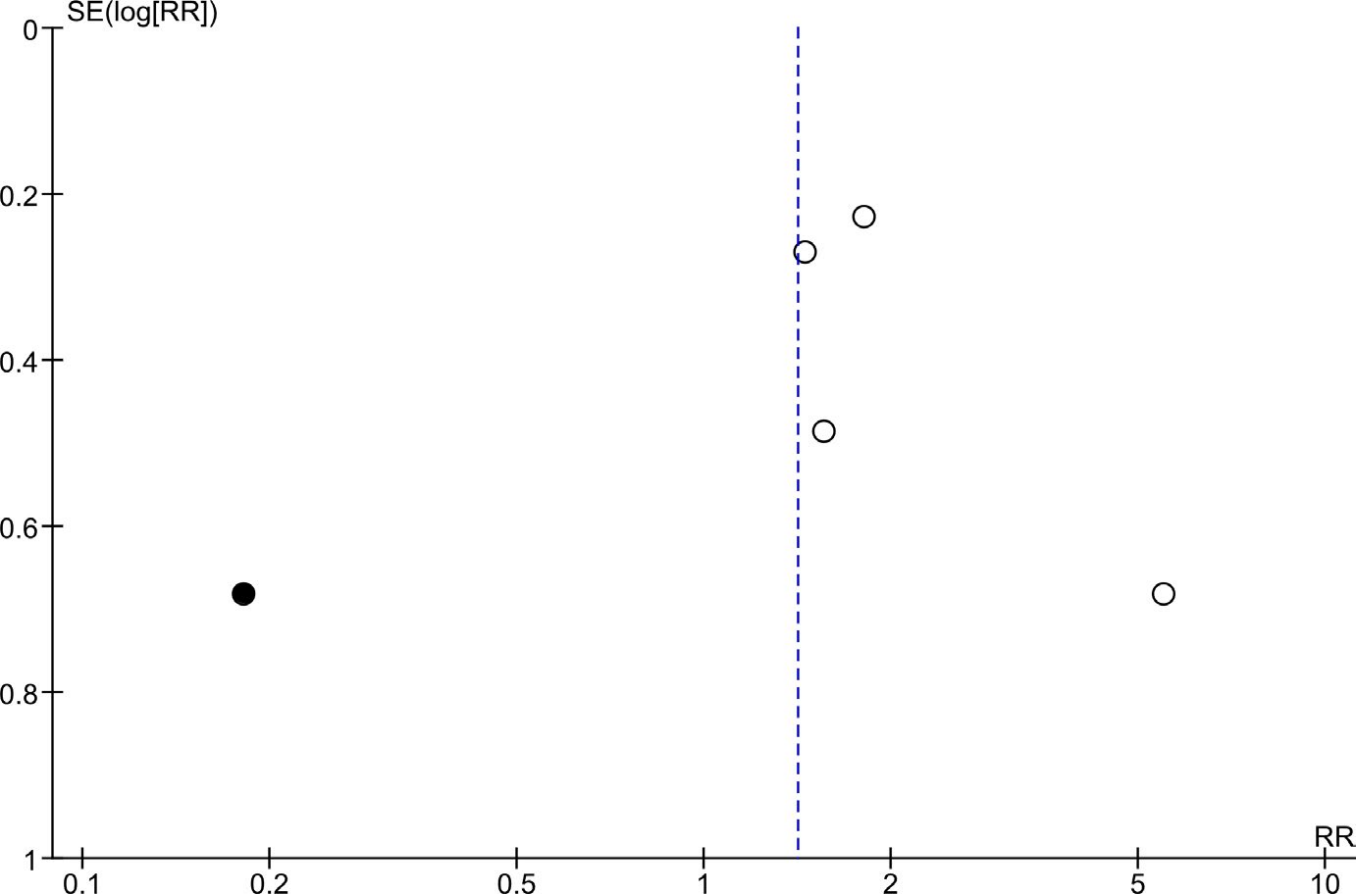
Funnel plots adjusted by missing study (black circles) of the left side of the mean effect. One study showed high mean effect of response, and we did trim and fill analysis of response rate adjust missing study (black circles) of the left side of the mean effect

**FIGURE 6:**
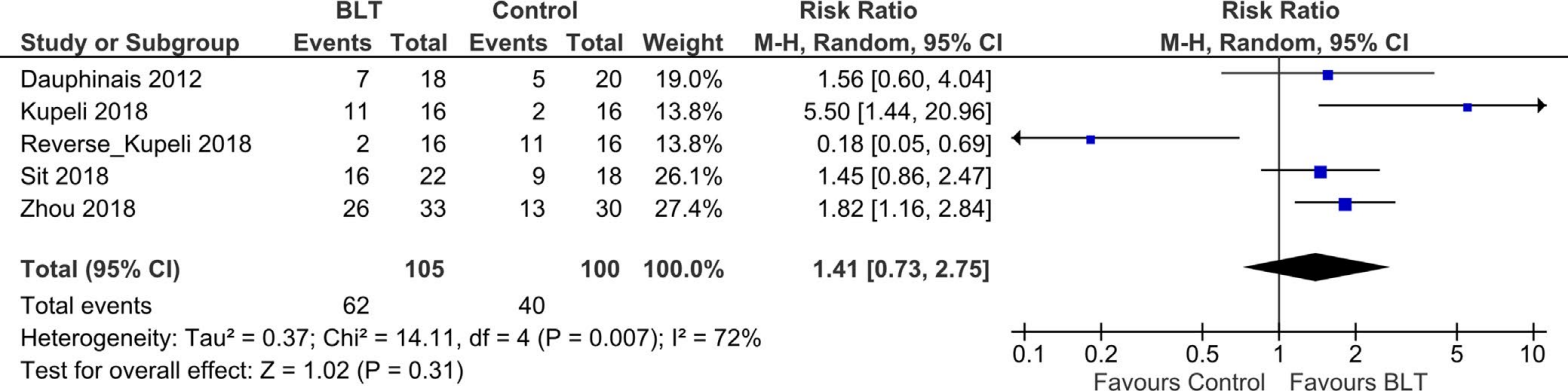
Efficacy of light therapy for bipolar depression with trim and fill analysis (response rate). By including the missing study, there is a possibility that the risk ratios changed 1.78 to 1.41 (95% CI 0.73–2.75, *p* = .31; *I* ^2^ = 72%). Also, value of *I* ^2^ changed 17% to 72% that indicates the degree of heterogeneity changed to moderately high

Four studies were included and a total of 190 participants with bipolar depression were evaluated for the effects of light therapy; 94 (49.5%) individuals were in the light therapy group and 96 (50.5%) individuals were in the control group. All studies included the response rate; however, one study did not mention about remission rate (Zhou et al., 2018). Figure 4 shows the results of a meta‐analysis that the number of patients achieving clinical response after light therapy. The meta‐analysis shows risk ratios of 1.78 (95% CI 1.24–2.56, *p* = .002; *I* ^2^ = 17%) demonstrating a significant effect of adjunctive light therapy in the response rate of patients with bipolar disorder. Number Needed to Treat (NNT) of response rate was 3.04. The value of *I* ^2^ indicates little variability between studies that cannot be explained by chance. The study of Yorguner Kupeli et al. 2018 showed high mean effect of response as showed in Figure 3, we did trim and fill analysis of response rate adjust missing study (black circles) of the left side of the mean effect (Figure 5). By including the missing study, there is a possibility that the risk ratios changed 1.78 to 1.41 (95% CI 0.73–2.75, *p* = .31; *I* ^2^ = 72%; Figure 6). Also, value of *I* ^2^ changed 17% to 72% that indicates the degree of heterogeneity changed to moderately high.

**FIGURE 7:**
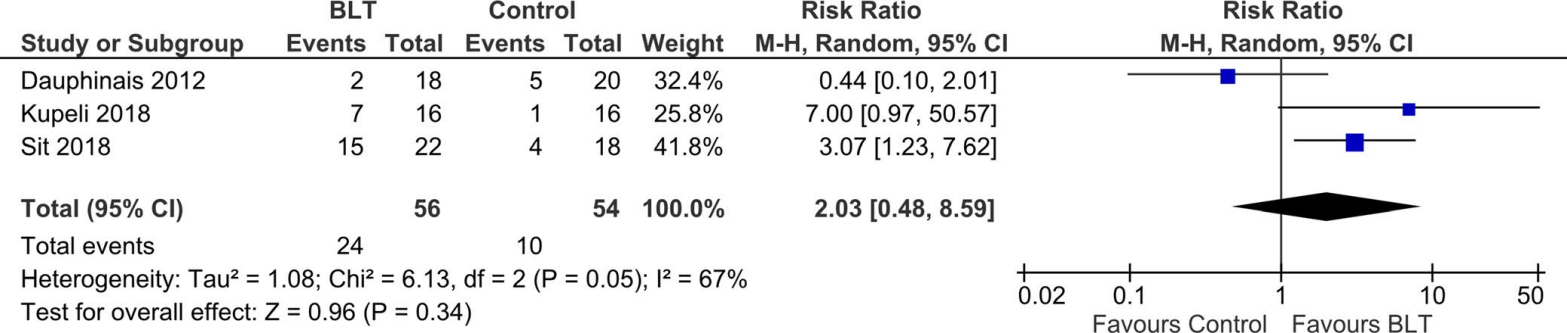
Efficacy of light therapy for bipolar depression (remission rate). The meta‐analysis shows risk ratios of 2.03 (95% CI 0.48–8.59, *p* = .34; *I* ^2^ = 67%) demonstrating no significant effect of light therapy in the remission rate of patients with bipolar disorder

The study of Zhou et al. did not mention about remission rate, we excluded their study to analyze the remission rate. Three studies were included and a total of 116 participants with bipolar depression were evaluated for the effects of light therapy; 57 (49.1%) individuals were in the light therapy group and 59 (50.9%) individuals were in the control group. Figure 6 shows the results of a meta‐analysis that the number of patients achieving remission after light therapy. The meta‐analysis shows risk ratios of 2.03 (95% CI 0.48–8.59, *p* = .34; *I* ^2^ = 67%) demonstrating no significant effect of light therapy in the remission rate of patients with bipolar disorder (Figure 7). However, the value of *I* ^2^ indicates degree of heterogeneity was moderately high. Number Needed to Treat (NNT) of remission rate was 4.11.

### 3.5 Acceptability and adverse effects

As shown in Table 1, throughout the included studies, we found that the dropout rate among those receiving bright light treatment was low and that it was considered well‐tolerated and none of the articles reported any serious adverse effects. We found that the dropout rate among those receiving bright light treatment was 13.8% (*n* = 13) and 21.9% (*n* = 21) in the control group. Manic switch rate was 1.1% (*n* = 1) in the light therapy group and 1.2% (*n* = 1) in the control group. Another adverse effects included studies were as follows: headache: 14.9% (*n* = 13), irritability: 4.26% (*n* = 4), sleep disturbance: 2.13% (*n* = 2), insomnia: 1.06% (*n* = 1), dizziness: 1.06% (*n* = 1), fatigue: 1.06% (*n* = 1), confusion and sedation: 1.06% (*n* = 1) in light therapy group, and headache: 12.5% (*n* = 12), irritability: 2.08% (*n* = 2), nightmare: 1.04% (*n* = 1), insomnia: 1.04% (*n* = 1), nausea: 1.04% (*n* = 1), and palpitation: 1.04% (*n* = 1) in control group.

## 4 DISCUSSION

The aim of this study was to investigate the adjunctive effects of light therapy for treating bipolar depression by using the data of RCT. We found that adjunctive light therapy is an effective treatment for reducing the depression symptoms among patients with bipolar depression. Previously meta‐analysis of Tseng et al. showed that augmentation treatment with light therapy significantly decreased disease severity of bipolar depression (Tseng et al., 2016). However, the value of *I* ^2^ was 70.1% which indicated significant heterogeneity was found among included studies in their analysis (Tseng et al., 2016). This study showed that a significant effect of adjunctive light therapy in the response rate of patients with bipolar disorder with little variability between studies (*I* ^2^ = 17%). Therefore, our result is superior to previous meta‐analysis in the point view of consistency of evidence. We also did trim and fill analysis of response rate adjust missing study, the result showed no significant effect of adjunctive light therapy for bipolar depression. However, value of *I* ^2^ changed 17% to 72% that indicates the degree of heterogeneity changed to moderately high. We found no significant effect of light therapy in the remission rate of patients with bipolar disorder. However, the value of *I* ^2^ (*I* ^2^ = 67%) indicates degree of heterogeneity was moderately high. Remission rate of adjunctive light therapy differ among included studies, study of Sit et al. reported 68.2% and study of Dauphinais et al. reported 11.1% respectively (Table 1). Therefore, further high quality RCT is needed to clarify the effect of adjunctive light therapy for bipolar depression. Our result showed BLT is an effective adjunctive treatment option for bipolar depression, the most efficient light parameters are not yet fully determined. Recent review of BLT for bipolar depression recommended that light intensity be below 10,000 lux, depending on the duration of light exposure (10,000 lux for 30 min per day or 5,000 lux for 1 hr per day, or 2,500 lux for 2 hr per day; Maruani & Geoffroy, 2019). Further, they suggested an increase in the duration of light exposure to prevent switching mania, for instance, 5,000 to 7,000 lux with an increase of 15min per week until 60 min (Maruani & Geoffroy, 2019; Sit et al., 2018). Among the four RCT we used for meta‐analysis, strategy of light therapy and treatment duration differed. Of these, the light treatment strategy of Zhou et al. (5,000 lux, 10,000 Kelvin, 60 min, morning, 2 weeks; Zhou et al., 2018) showed the highest response rate (78.7%) without switching mania (Table 1). Thus, this strategy might be the most effective way for acute‐phase treatment of bipolar depression.

Further, our findings regarding dropout rates indicate that light therapy was well‐tolerated compared with those in the control groups. Manic switch rate of our study was 1.1% in the light therapy group, which is lower than 2.3% that reported in previous studies (Benedetti, 2018). The light treatment strategy of Sit et al. was dose‐titration protocol in the midday as a precaution against inducing hypomania or mixed symptoms. Rest of three studies were full daily light dose (5,000 – 10,000 lux) in the morning. Our results suggest that light therapy in the morning might be safe with regard to polarity shifting. In addition, none of the articles reported any serious adverse effects and most common side effects was headache (14.9%). Therefore, we recommend light therapy as a safe adjunctive treatment option for bipolar patients especially for those who have had episodes of manic switches through the use of antidepressant drugs or patients who preferred nonpharmacological treatments such as pregnant women and older adults.

In this review, we excluded the studies that combined sleep deprivation with light therapy (Benedetti et al., 2003, 2005, 2007, 2009, 2010, 2014; Chojnacka, et al., 2016; Colombo et al., 2000; Sikkens et al., 2019; Suzuki et al., 2016, 2018; Vai et al., 2015; Wu et al., 2009) to evaluate the effect of adjunctive light therapy itself. Recent meta‐analysis found that sleep deprivation is effective for the acute treatment of bipolar depression (Boland et al., 2017). In addition, adjunctive treatment with bright light therapy and sleep deprivation is effective for bipolar depression (Tseng et al., 2016). Furthermore, the effect of light therapy with total sleep deprivation could have rapid response speed (Benedetti, et al., 2014), combination of light therapy and sleep deprivation could be effective in some bipolar depression patients. However these combined treatments make discernment of individual contributions to response difficult. Future studies are needed to separate the effect of sleep deprivation and light therapy.

Dawn simulation is another light treatment application, in which light exposure is increased from 0 to 200–300 lux over 1.0–2.5 hr like “dawn” (Golden et al., 2005). There was no study of dawn simulation for treating bipolar depression; however an open‐label study found 400 lux (low intensity light) for 2 hr could be effective for treating bipolar depression (Deltito et al., 1991). Further, the study by Sit et al. (2018) showed that the response rate and remission rate in the control group with exposure to light intensity of only 50 lux for 15‐60min were 50.0% and 22.2%, respectively. This study suggested that low intensity light might have therapeutic effect for treating bipolar depression although it was weaker than that in BLT (Sit et al., 2018). Meta‐analyses studies revealed that there is a significant antidepressant effect in dawn simulation for seasonal affective disorder (Geoffroy et al., 2014). Rosenthal et al. (1984) described that most of SAD patients had a bipolar affective disorder, especially bipolar II. Bipolar disorder showed seasonal fluctuations in mood and behavior (Geoffroy et al., 2014). In addition, being in rooms with eastern windows, which received direct sunlight in the morning (i.e., natural dawn simulation), reduced the days of hospital stay for those with bipolar depression (Benedetti et al., 2001). Therefore, dawn simulation might be effective for treating bipolar depression.

Light directly affects the mood by having an antidepressant effect such as that seen in light therapy and has an anti‐manic effect on deprivation such as that seen in dark therapy and virtual darkness therapy (blue light‐blocking treatment by means of orange‐tinted glasses; Barbini et al., 2005; Henriksen et al., 2016). We recently reported two cases of bipolar II patients with hypomania who responded to treatment with gray sunglasses used in the daytime (Shirahama et al., 2019). Wirz‐Justice and colleagues reported an interesting case on a refractory bipolar I rapid‐cycling patient, who failed to be treated with only a mood stabilizer, and yet improved by the addition of a combination of dark therapy and daytime light therapy (Wirz‐Justice et al., 1999). A more convenient approach, as we previously propounded, is the “light modulation therapy” which is a combination of BLT for depressive mood and sunglasses therapy (i.e., deprivation of environment light) for hypomanic/manic mood of bipolar patients as the adjunctive treatment (Hirakawa et al., 2019; Terao & Hirakawa, 2015). Specifically, when bipolar patients feel depressed or sad, we recommend them to increase ambient light exposure by opening the curtains in the morning or walking outside. When the patients feel uplifted, we recommend that they reduce their ambient light exposure by turning down the room light or by wearing sunglasses. A recent cohort study investigated the association between daytime light exposure under real‐life situations and depressive symptoms in bipolar disorder and found that greater daytime light exposure in daily life is associated with decreased depressive symptoms (Esaki et al., 2019). We think making a good use of light is beneficial for patients with bipolar disorder. Further RCT studies of light therapy (BLT and dawn simulation) and dark therapy (dark therapy and sunglasses therapy) are needed for further clinical evidence.

### 4.1 Limitations

There are some limitations to this study: first, the small number of RCTs and small sample sizes in each. Second, light treatment strategy differed among studies such as light intensities, light exposure durations, and daily timing. Third, the BLT of this meta‐analysis was adjunctive therapy and most of the patients included in the studies were treated with mood stabilizers or antidepressants. We could not remove the effect of drugs or the interaction of drug and BLT.

## 5 CONCLUSION

This systematic review and meta‐analysis suggest that adjunctive light therapy is an effective treatment for reducing depression symptoms for patients with bipolar depression. Moreover, this review indicates that there were no serious adverse effects and manic switch rate induced by light therapy. However, this review secured very limited amount of studies with RCT; therefore, more studies investigating light therapy for treating bipolar depression are needed in the future.

## CONFLICT OF INTEREST

None.

## AUTHOR CONTRIBUTIONS

For a systematic review, the search was conducted by two independent authors (HH and MM). TT performed checks to ensure quality and consistency of the assessment and made the final judgment and decision. HH analyzed the data and wrote the manuscript. MM and NI and TT provided constructive criticism it. All authors reviewed the manuscript and made contributions to it.

### Peer Review

The peer review history for this article is available at https://publons.com/publon/10.1002/brb3.1876.

## Data Availability

The datasets generated and analyzed during the current study are available from the corresponding author on reasonable request.
